# Prevalence and associated factors for workplace violence among general practitioners in China: a national cross-sectional study

**DOI:** 10.1186/s12960-022-00736-x

**Published:** 2022-05-16

**Authors:** Jing Feng, Zihui Lei, Shijiao Yan, Heng Jiang, Xin Shen, Yanling Zheng, Minyi Yu, Xin Meng, Hongkun Di, Wenqi Xia, Ying Zhou, Tingting Yang, Cheng Su, Fanjun Cheng, Zuxun Lu, Yong Gan

**Affiliations:** 1 Department of Social Medicine and Health Management, School of Public Health, Tongji Medical College, Huazhong University of Science and Technology, Wuhan, Hubei China; 2 Department of Emergency Medicine, Hunan Provincial People’s Hospital/The First Affiliated Hospital, Hunan Normal University, Changsha, Hunan China; 3 Key Laboratory of Emergency and Trauma of Ministry of Education, Hainan Medical University, Haikou, Hainan China; 4 Centre for Alcohol Policy Research, School of Psychology and Public Health, La Trobe University, Melbourne, VIC Australia; 5 Centre for Health Equity, Melbourne School of Population and Global Health, University of Melbourne, Melbourne, VIC Australia; 6 Shouyilu Street Community Health Service Center, Wuhan, Hubei China; 7 Department of Nutrition, Henan Provincial People’s Hospital and Zhengzhou University People’s Hospital, Zhengzhou, Henan China; 8 Union Hospital, Tongji Medical College, Huazhong University of Science and Technology, Wuhan, Hubei China

**Keywords:** General practitioners, Workplace violence, Primary health care, China

## Abstract

**Background:**

General practitioners (GPs) were at risk of violence in their everyday working lives. Workplace violence (WPV) among GPs is a global public health concern. This study aimed to investigate the prevalence and factors associated with WPV among GPs in China.

**Methods:**

A cross-sectional study was conducted among 4376 GPs in eastern, central, and western China between March and May 2021 using a structured self-administered questionnaire. The multivariable stepwise logistic regression model was used to examine the factors associated with WPV among GPs in China.

**Results:**

Among these respondents, 14.26% of them reported exposure to WPV in the past 12 months. GPs who were female, practised in a rural area, made home visits occasionally, worked in a fair or good practice environment or work environment, and had a fair or good relationship with patients were less likely to encounter any type of WPV. In addition, GPs who served patients over 20 per day and worked overtime occasionally or frequently were more likely to be exposed to WPV. The determinants of WPV varied in different types of WPV and sexes.

**Conclusions:**

The prevalence of WPV among GPs is low in China. Our findings could inform the measures to reduce the WPV among GPs.

## Background

Workplace violence (WPV) is defined as “incidents where staff are abused, threatened, or assaulted in circumstances related to their work, including commuting to and from work, involving an explicit or implicit challenge to their safety, well-being or health” [1]. While WPV affects practically all sectors and all categories of workers, the health sector is at major risk [1, 2]. Health care professionals, including general practitioners (GPs), are at risk of violence in their everyday working lives [2]. The World Health Organization (WHO) has reported that between 8 and 38% of health workers suffer physical violence at some point in their careers, and many more are threatened or exposed to verbal aggression [3]. WPV among healthcare workers has adverse effects on individuals, organizations, and societies [4–7]. Since GPs are key providers of community health services and the backbone of the primary healthcare system, the quality of a nation’s healthcare system is linked to the efficiency and effectiveness of its GPs workforce. Therefore, the primary prevention of WPV in GPs should be considered a public health priority.

Studies investigating WPV in GPs have been conducted in many developed countries [2, 8–20]. Associations between age [2, 9, 13, 15], sex [2, 9, 12, 13, 15], practice setting [2, 9, 12], working hours [2, 12, 15], years in practice [2, 9, 15], full or part-time [9], and home visits [9, 15] and WPV among GPs have been investigated in the previous studies. A few research studies have investigated the prevalence and factors associated with WPV among GPs in China [6, 7, 21, 22]. However, the sample size (ranging from 442 to 1015), practice setting (only township hospitals or one province), and influencing factors associated with WPV (mainly socio-demographic factors) investigated in these studies were limited. Thus, this study aimed to conduct a more comprehensive survey to investigate WPV and its determinants among Chinese GPs at the national level. Additionally, the characteristics, reasons, and reactions to WPV and gender differences of influencing factors in different types of WPV among GPs were analyzed in our study.

## Methods

### Study population

A national cross-sectional study was conducted between March and May 2021 in China. A multi-stage stratified random sampling strategy was used to obtain research samples. First, five provincial administrative regions were randomly selected from each of three different levels of economic development and geographical regions, eastern China (Shanghai, Liaoning, Zhejiang, Guangdong, and Fujian), central China (Hubei, Hunan, Shanxi, Anhui, and Henan), and western China (Chongqing, Sichuan, Shaanxi, Yunnan, and Guangxi Zhuang Autonomous Region). Second, 40 community health service institutions were randomly selected from each province. Third, 40% of on-post GPs with 1 year or more of work experience were randomly selected from each community health service institution to complete a self-administered questionnaire through WeChat. A total of 4632 GPs were invited to participate in this survey. The 256 questionnaires were excluded because of logical errors, and 4376 were identified as eligible questionnaires and used in this analysis, yielding a response rate of 94.47%.

The study protocol was approved by the Ethics Committee of the Tongji Medical College Institutional Review Board, Huazhong University of Science and Technology, Wuhan, China (no. [2021] IEC (S099)). Informed consent was obtained from all survey participants.

### Questionnaire design and content

The questionnaire was designed based on a literature review, group discussions, and mock interviews. It included nine parts: socio-demographic information, work-related factors, perceived over-qualification, professional identity, WPV, depression symptoms, career orientation, psychological capital, and intention to stay. Socio-demographic data were region, age, sex, ethnicity, marital status, education level, and annual personal income. Work-related factors included work tenure, practice setting, contract status, professional title, management responsibility, weekly working hours, daily consultation numbers, consultation length per capita, administrative task, working overtime, frequency of home visits, workload, occupational stress, occupational development opportunities, work environment, the relationship between colleagues, recognition by residents, physician–patient relations, and practice environment. The WPV section collected the prevalence, reasons, and characteristics of WPV. Given the purpose of this study, the data from sections 1, 2, and 5 were included.

### Measurement

WPV was evaluated by the Chinese version of the Workplace Violence Scale developed by Wang et al. [23]. The scale has good reliability and validity for measuring the incidence of WPV when applied to medical staff in China [6, 24]. It includes 5 items measured with a four-point Likert scale ranging from 0 (never) to 3 (more than 3 times/year). In this study, the Cronbach’s alpha for WPV was 0.81. WPV was classified as physical (physical assault and physical sexual assault) and non-physical (verbal abuse, threats, and verbal sexual harassment) violence. Moreover, the GPs reported their experience of WPV in the past year before taking the survey.

### Data collection and quality control

To improve the quality of the questionnaire, a pre-test was conducted in Wuhan’s community health centres (CHCs). An online questionnaire designed using the software Questionnaire Star was distributed to the GPs through WeChat. To prevent duplicate records from the same participant, each device (e.g., smartphone or computer) was limited to complete the questionnaire once. Data were entered into a web-based database by trained investigators to ensure accuracy.

### Data analysis

All analyses were performed using Statistical Package for Social Sciences (SPSS, Inc., Chicago, IL, Version 26.0). The descriptive statistics for the categorical variables were reported as the frequency (percentage). Multivariable stepwise logistic regression model was used to examine potential impact factors for WPV among GPs. The dependent variables, including any type of violence, physical violence, and non-physical violence, were treated as categorical variables. The predictive variables included all socio-demographic characteristics and work-related factors of GPs. The multivariable stepwise logistic regression analysis (cutoffs for selection and elimination: *P* = 0.05 and *P* = 0.10, respectively) was used to calculate the odds ratios (ORs) and 95% confidence intervals (CIs) for factors associated with the prevalence of WPV toward GPs. A value of *P* < 0.05 (two-tailed) was considered statistically significant.

## Results

Among the respondents, nearly half of GPs (46.07%) were from the eastern region, and 27.06% and 26.87% of GPs were from central and western China, respectively. The mean age of respondents was 40.64 [standard error (SD) = 8.62, ranging from 21 to 75 years]. More than half were females. The majority of respondents were Han Chinese, married, had a bachelor’s degree, and had an annual personal income lower than 100,000 ¥ (Table 1). Most of GPs practised in urban areas, had a permanent contract, and had no management responsibility. Nearly half of GPs worked less than 10 years, had intermediate professional titles, and worked less than 40 h a week. More details of work-related factors of GPs are shown in Table 2.

**Table 1:** Distributions of demographic characteristics of WPV in general practitioners

Variable	Total *n* (%)	Any type of WPV^*^ *n* (%)	Physical violence *n* (%)	Non-physical violence *n* (%)
Total	4376 (100.00)	624 (100.00)	236 (100.00)	614 (100.00)
Region				
Eastern China	2016 (46.07)	300 (48.08)	110 (46.61)	296 (48.21)
Central China	1184 (27.06)	184 (29.49)	69 (29.24)	181 (29.48)
Western China	1176 (26.87)	140 (22.44)	57 (24.15)	137 (22.31)
Age (years)				
< 30	471 (10.76)	64 (10.26)	22 (9.32)	63 (10.26)
30–	1509 (34.48)	228 (36.54)	73 (30.93)	226 (36.81)
40–	1684 (38.48)	244 (39.10)	102 (43.22)	240 (39.09)
≥ 50	712 (16.27)	88 (14.10)	39 (16.53)	85 (13.84)
Sex				
Male	1778 (40.63)	321 (51.44)	149 (63.14)	314 (51.14)
Female	2598 (59.37)	303 (48.56)	87 (36.86)	300 (48.86)
Ethnicity				
Han	4065 (92.89)	587 (94.07)	219 (92.80)	579 (94.30)
Others	311 (7.11)	37 (5.93)	17 (7.20)	35 (5.70)
Marital status				
Unmarried/widowed/divorced	564 (12.89)	79 (12.66)	28 (11.86)	77 (12.54)
Married	3812 (87.11)	545 (87.34)	208 (88.14)	537 (87.46)
Education level				
Associate’s degree or vocational diploma	1103 (25.21)	120 (19.23)	53 (22.46)	112 (18.24)
Bachelor degree	2974 (67.96)	455 (72.92)	169 (71.61)	453 (73.78)
Master degree or higher	299 (6.83)	49 (7.85)	14 (5.93)	49 (7.98)
Annual personal income (¥)				
< 100,000	2930 (66.96)	410 (65.71)	159 (67.37)	400 (65.15)
100,000–	1001 (22.87)	141 (22.60)	55 (23.31)	141 (22.96)
≥ 150,000	445 (10.17)	73 (11.70)	22 (9.32)	73 (11.89)

*WPV* workplace violence

^∗^Includes those who experienced only physical, only non-physical, or both types of workplace violence

**Table 2:** Distributions of work-related factors of WPV in general practitioners

Variable	Total *n* (%)	Any type of WPV^*^ *n* (%)	Physical violence *n* (%)	Non-physical violence *n* (%)
Total	4376 (100.00)	624 (100.00)	236 (100.00)	614 (100.00)
Work tenure (years)				
< 10	1984 (45.34)	270 (43.27)	92 (38.98)	263 (42.83)
10–	1442 (32.95)	203 (32.53)	86 (36.44)	201 (32.74)
≥ 20	950 (21.71)	151 (24.20)	58 (24.58)	150 (24.43)
Practice setting				
Urban	3335 (76.21)	501 (80.29)	182 (77.12)	494 (80.46)
Rural	1041 (23.79)	123 (19.71)	54 (22.88)	120 (19.54)
Contract status				
Temporary	1072 (24.50)	139 (22.28)	51 (21.61)	136 (22.15)
Permanent	3304 (75.50)	485 (77.72)	185 (78.39)	478 (77.85)
Professional title				
Elementary or below	1647 (37.64)	199 (31.89)	71 (30.08)	192 (31.27)
Intermediate	1964 (44.88)	299 (47.92)	117 (49.58)	296 (48.21)
Senior	765 (17.48)	126 (20.19)	48 (20.34)	126 (20.52)
Management responsibility				
Yes	1048 (23.95)	162 (25.96)	69 (29.24)	158 (25.73)
No	3328 (76.05)	462 (74.04)	167 (70.76)	456 (74.27)
Weekly working hours				
≤ 40	2018 (46.12)	246 (39.42)	87 (36.86)	242 (39.41)
40–	1324 (30.26)	189 (30.29)	60 (25.42)	186 (30.29)
> 50	1034 (23.63)	189 (30.29)	89 (37.71)	186 (30.29)
Daily consultation numbers				
< 20	1322 (30.21)	130 (20.83)	54 (22.88)	126 (20.52)
20–	1649 (37.68)	215 (34.46)	86 (36.44)	211 (34.36)
≥ 40	1405 (32.11)	279 (44.71)	96 (40.68)	277 (45.11)
Consultation length per capita (minutes)				
≤ 10	2942 (67.23)	456 (73.08)	159 (67.37)	450 (73.29)
10–	1053 (24.06)	124 (19.87)	52 (22.03)	121 (19.71)
> 20	381 (8.71)	44 (7.05)	25 (10.59)	43 (7.00)
Administrative task (% total workload)				
≤ 10	2671 (61.01)	349 (55.93)	124 (52.54)	344 (56.03)
11–	810 (18.51)	122 (19.55)	45 (19.07)	120 (19.54)
> 20	895 (20.45)	153 (24.52)	67 (28.39)	150 (24.43)
Working overtime				
Never	81 (1.85)	2 (0.32)	1 (0.42)	2 (0.33)
Occasion	2058 (47.03)	206 (33.01)	66 (27.97)	201 (32.74)
Frequent	2237 (51.12)	416 (66.67)	169 (71.61)	411 (66.94)
Home visit				
Never	210 (4.80)	39 (6.25)	19 (8.05)	39 (6.35)
Occasion	1525 (34.85)	187 (29.97)	71 (30.08)	182 (29.64)
Frequent	2641 (60.35)	398 (63.78)	146 (61.86)	393 (64.01)
Workload				
Low	43 (0.98)	3 (0.48)	2 (0.85)	3 (0.49)
Intermediate	1405 (32.11)	118 (18.91)	36 (15.25)	116 (18.89)
High	2928 (66.91)	503 (80.61)	198 (83.90)	495 (80.62)
Occupational stress				
Low	74 (1.69)	8 (1.28)	3 (1.27)	8 (1.30)
Intermediate	1488 (34.00)	122 (19.55)	35 (14.83)	120 (19.54)
High	2814 (64.31)	494 (79.17)	198 (83.90)	486 (79.15)
Occupational development opportunities				
Fewer	1732 (39.58)	319 (51.12)	124 (52.54)	315 (51.30)
General	2116 (48.35)	254 (40.71)	88 (37.29)	250 (40.72)
More	528 (12.07)	51 (8.17)	24 (10.17)	49 (7.98)
Work environment				
Bad	644 (14.72)	168 (26.92)	70 (29.66)	165 (26.87)
Fair	2368 (54.11)	327 (52.40)	118 (50.00)	321 (52.28)
Good	1364 (31.17)	129 (20.67)	48 (20.34)	128 (20.85)
Relationship between colleagues				
Bad	23 (0.53)	11 (1.76)	8 (3.39)	11 (1.79)
Fair	636 (14.53)	108 (17.31)	59 (25.00)	106 (17.26)
Good	3717 (84.94)	505 (80.93)	169 (71.61)	497 (80.94)
Recognition by residents				
Low	772 (17.64)	179 (28.69)	64 (27.12)	175 (28.50)
Intermediate	2194 (50.14)	311 (49.84)	119 (50.42)	306 (49.84)
High	1410 (32.22)	134 (21.47)	53 (22.46)	133 (21.66)
Physician–patient relations				
Bad	684 (15.63)	226 (36.22)	90 (38.14)	223 (36.32)
Fair	2088 (47.71)	297 (47.60)	110 (46.61)	293 (47.72)
Good	1604 (36.65)	101 (16.19)	36 (15.25)	98 (15.96)
Practice environment				
Bad	903 (20.64)	252 (40.38)	99 (41.95)	247 (40.23)
Fair	2471 (56.47)	305 (48.88)	116 (49.15)	301 (49.02)
Good	1002 (22.90)	67 (10.74)	21 (8.90)	66 (10.75)

*WPV* workplace violence

^∗^Includes those who experienced only physical, only non-physical, or both types of workplace violence

### Prevalence of WPV

Table 3 shows the prevalence of five types of violence in Chinese GPs in the past 12 months. Verbal abuse was the most common type of violence (13.44%), followed by threat (9.23%), verbal sexual harassment (4.68%), physical assault (4.59%), and physical sexual assault (2.29%). The prevalence of any type of WPV, non-physical violence, and physical violence was 14.26%, 14.03%, and 5.39%, respectively.

**Table 3:** Frequency of five types of violence among general practitioners

Type of violence	Once *n* (%)	Two or three times *n* (%)	More than three times *n* (%)	Total *n* (%)
Physical assault	121 (2.77)	60 (1.37)	20 (0.46)	201 (4.59)
Verbal abuse	153 (3.50)	199 (4.55)	236 (5.39)	588 (13.44)
Threat	191 (4.36)	122 (2.79)	91 (2.08)	404 (9.23)
Verbal sexual harassment	93 (2.13)	55 (1.26)	57 (1.30)	205 (4.68)
Physical sexual assault	56 (1.28)	25 (0.57)	19 (0.43)	100 (2.29)

### Reasons and characteristics of WPV

Table 4 shows statistics of the characteristics and reasons for the latest WPV in the last 12 months. We found that the main perpetrators of WPV toward to GPs were patients and 79.49% of perpetrators were males. The WPV was more prevalent among respondents who worked morning shifts and most of the WPV incidents occurred in GPs’ offices. In addition, the top three reasons for causing WPV were “unmet patients’ needs”, “long waiting times”, and “being unsatisfied with GPs’ service”. The top three corresponding actions were “sought help from their managers”, “took no action”, and “stopped the perpetrations”.

**Table 4:** Characteristics, reasons, and reactions to WPV among general practitioners

Variable	Any type of WPV^*^ *n* (%)	Physical violence *n* (%)	Non-physical violence *n* (%)
Total	624 (100.00)	236 (100.00)	614 (100.00)
Perpetrators			
Patients	316 (50.64)	102 (43.22)	313 (50.98)
Patients’ families	252 (40.38)	111(47.03)	245 (39.90)
Colleagues	8 (1.28)	5 (2.12)	8 (1.30)
Managers/Supervisors	3 (0.48)	0 (0.00)	3 (0.49)
External colleagues	1 (0.16)	0 (0.00)	1 (0.16)
General public	20 (3.21)	9 (3.81)	20 (3.26)
Visitors	11 (1.76)	6 (2.54)	11 (1.79)
Others	13 (2.08)	3 (1.27)	13 (2.12)
Sex of perpetrators			
Male	496 (79.49)	187 (79.24)	487 (79.32)
Female	128 (20.51)	49 (20.76)	127 (20.68)
Age of perpetrators (years)			
< 30	26 (4.17)	13 (5.51)	24 (3.91)
30–	86 (13.78)	42 (17.80)	84 (13.68)
40–	127 (20.35)	55 (23.31)	125 (20.36)
50–	185 (29.65)	68 (28.81)	183 (29.80)
≥ 60	198 (31.73)	57 (24.15)	196 (31.92)
Time of violence			
Morning shifts	331 (53.04)	103 (43.64)	326 (53.09)
Afternoon shifts	158 (25.32)	56 (23.73)	156 (25.41)
Night shifts	101 (16.19)	60 (25.42)	100 (16.29)
After hours	34 (5.45)	17 (7.20)	32 (5.21)
Settings of violence			
Wards	31 (4.97)	18 (7.63)	29 (4.72)
GPs’ office	468 (75.00)	181 (76.69)	464 (75.57)
Nurse station	25 (4.01)	9 (3.81)	24 (3.91)
On the road off work	5 (0.80)	2 (0.85)	5 (0.81)
Others	95 (15.22)	26 (11.02)	92 (14.98)
Reasons for violence			
Long waiting times	208 (33.33)	75 (31.78)	206 (33.55)
Dissatisfied with GPs’ service	175 (28.04)	67 (28.39)	171 (27.85)
Unmet patient needs	447 (71.63)	167 (70.76)	444 (72.31)
Patients’ death	22 (3.53)	10 (4.24)	21 (3.42)
Perpetrators’ mental disorder	56 (8.97)	26 (11.02)	54 (8.79)
Thinking medical costs high	136 (21.79)	55 (23.31)	134 (21.82)
Requiring financial compensation	49 (7.85)	24 (10.17)	48 (7.82)
After taking drugs/drinking	86 (13.78)	44 (18.64)	84 (13.68)
Dissatisfied with treatment effect	117 (18.75)	46 (19.49)	117 (19.06)
Others	99 (15.87)	39 (16.53)	98 (15.96)
Reactions to violence			
Took no action	197 (31.57)	67 (28.39)	196 (31.92)
Tried to pretend it never happened	91 (14.58)	40 (16.95)	91 (14.82)
Stopped the perpetrators	187 (29.97)	67 (28.39)	183 (29.80)
Told friends/families	59 (9.46)	22 (9.32)	57 (9.28)
Told colleagues	153 (24.52)	61 (25.85)	153 (24.92)
Sought help from managers	206 (33.01)	80 (33.90)	206 (33.55)
Sought help from union	35 (5.61)	20 (8.47)	34 (5.54)
Called the police	111 (17.79)	69 (29.24)	105 (17.10)
Transferred to another position	5 (0.80)	3 (1.27)	5 (0.81)
Completed a WPV report	71 (11.38)	29 (12.29)	71 (11.56)
Prosecuted	4 (0.64)	3 (1.27)	4 (0.65)
Others	64 (10.26)	22 (9.32)	64 (10.42)

*WPV* workplace violence, *GPs* general practitioners

^∗^Includes those who experienced only physical, only non-physical, or both types of workplace violence

### Factors associated with WPV

Table 5 shows the results of multivariable stepwise logistic regression analysis. GPs who were female (OR = 0.67), practised in the rural area (OR = 0.67), did home-visiting occasionally (OR = 0.62), worked in fair (OR = 0.68) or good (OR = 0.62) environments, had a fair (OR = 0.49) or good (OR = 0.23) relationship with patients, and worked in a fair (OR = 0.66) or good (OR = 0.64) practice environment were less likely to experience any type of WPV. By contrast, GPs who served over 20 patients per day (20–: OR = 1.38; ≥ 40: OR = 2.11) and occasionally (OR = 4.27) or frequently (OR = 6.30) worked overtime were more likely to be exposed to any type of WPV.

**Table 5:** Logistic stepwise regression analysis of associated factors for WPV among Chinese general practitioners

Variable	Any type of WPV^*a^	Physical violence^b^	Non-physical violence^c^
Sex (ref. Male)			
Female	0.67 (0.55–0.80)	0.46 (0.35–0.62)	0.65 (0.54–0.79)
Education level (ref. Associate’s degree or vocational diploma)			
Bachelor degree	–	–	1.37 (1.08–1.74)
Master degree or higher	–	–	1.72 (1.15–2.57)
Practice setting (ref. Urban)			
Rural	0.67 (0.53–0.84)	–	0.68 (0.54–0.87)
Daily consultation numbers (ref. < 20)			
20–	1.38 (1.08–1.76)	–	1.37 (1.07–1.76)
≥ 40	2.11 (1.65–2.69)	1.67 (1.17–2.39)	2.11 (1.64–2.70)
Work overtime (ref. Never)			
Occasion	4.27 (1.01–17.99)	–	–
Frequent	6.30 (1.50–26.51)	–	5.98 (1.42–25.28)
Home visit (ref. Never)			
Occasion	0.62 (0.41–0.94)	–	0.63 (0.41–0.95)
Frequent	–	–	–
Occupational development opportunities (ref. Low)			
Middle	–	–	0.81 (0.66–0.98)
High	–	–	0.70 (0.49–0.99)
Work environment (ref. Bad)			
Fair	0.68 (0.53–0.86)	0.65 (0.47–0.91)	0.70 (0.55–0.89)
Good	0.62 (0.46–0.84)	–	0.69 (0.50–0.94)
Relationship between colleagues (ref. Bad)			
Fair	–	0.31 (0.12–0.82)	–
Good	–	0.17 (0.07–0.44)	–
Physician–patient relations (ref. Bad)			
Fair	0.49 (0.39–0.61)	0.49 (0.36–0.68)	0.49 (0.39–0.61)
Good	0.23 (0.17–0.32)	0.25 (0.16–0.38)	0.23 (0.17–0.32)
Practice environment (ref. Bad)			
Fair	0.66 (0.53–0.83)	–	0.69 (0.55–0.87)
Good	0.64 (0.44–0.93)	–	–

*WPV* workplace violence

^∗^Includes those who experienced only physical, only non-physical, or both types of workplace violence

^a^Adjustment for sex (male, female), practice setting (urban, rural), daily consultation numbers (< 20, 20–, ≥ 40), working overtime (never, occasion, frequent), home visit (never, occasion, frequent), work environment (bad, fair, good), physician–patient relations (bad, fair, good), and practice environment (bad, fair, good), which were included in the final model during the stepwise process

^b^Adjustment for sex (male, female), daily consultation numbers (< 20, 20–, ≥ 40), work environment (bad, fair, good), relationship between colleagues (bad, fair, good), and physician–patient relations (bad, fair, good), which were included in the final model during the stepwise process

^c^Adjustment for sex (male, female), education level (associate’s degree or vocational diploma, bachelor degree, master degree or higher), practice setting (urban, rural), daily consultation numbers (< 20, 20–, ≥ 40), working overtime (never, occasion, frequent), home visit (never, occasion, frequent), occupational development opportunities (fewer, general, more), work environment (bad, fair, good), physician–patient relations (bad, fair, good), and practice environment (bad, fair, good), which were included in the final model during the stepwise process

Compared with results for any type of WPV, more influencing factors for non-physical violence were found. Except for the factors mentioned above, GPs who had higher education levels (bachelor degree: OR = 1.37; master degree or higher: OR = 1.72) were more likely to experience non-physical violence. And GPs who had more opportunities for occupational development (middle: OR = 0.81; high: OR = 0.70) were less likely to be exposed to non-physical violence.

Intriguingly, having a fair (OR = 0.31) or good (OR = 0.17) colleague relationship could decrease the risk of experiencing physical violence. Furthermore, the logistic regression results reveal sex, daily consultation numbers, work environment, and physician–patient relations were significantly associated with all three types of violence.

Table 6 shows the results of GPs’ multivariable stepwise logistic regression analysis by sex. The results indicated that female GPs who worked in rural areas (OR = 0.57), being in fair (OR = 0.57) or good (OR = 0.65) work environments, had a fair (OR = 0.43) or good (OR = 0.23) relationship with patients, and worked in fair (OR = 0.68) or good (OR = 0.56) practice environments were less likely to be exposed to any type of WPV. However, female GPs serving more than 40 patients per day (OR = 2.52) were more likely to experience any type of WPV. Compared to female GPs, males were less likely to experience any type of WPV when they did home-visiting occasionally (OR = 0.46). Interestingly, the factors associated with female physical violence were interpersonal relationships, including a fair (OR = 0.17) or good (OR = 0.09) colleague relationship, and fair (OR = 0.38) or good (OR = 0.24) physician–patient relations. However, other work-related factors and all demographic characteristics were not statistically significantly associated with the prevalence of physical violence in females. Moreover, the education level, practice settings, daily consultation numbers, occupational development opportunities, work environment, and physician–patient relations were significantly associated with the prevalence of non-physical violence in females.

**Table 6:** Gender-stratified logistic stepwise regression analysis of associated factors for WPV among Chinese general practitioners

Variable	Male			Female		
	Any type of WPV^*a^	Physical violence^b^	Non-physical violence^c^	Any type of WPV^*d^	Physical violence^e^	Non-physical violence^f^
Education level (ref. Associate’s degree or vocational diploma)						
Bachelor degree	–	–	–		–	1.68 (1.14–2.47)
Master degree or higher	–	–	–		–	2.11 (1.22–3.67)
Practice setting (ref. Urban)						
Rural	0.70 (0.52–0.94)	–	0.69 (0.51–0.93)	0.57 (0.37–0.86)	–	0.59 (0.38–0.89)
Weekly working hours (ref. < 40)						
40–	–	0.50 (0.30–0.81)	–	–	–	–
≥ 50	–	–	–	–	–	–
Daily consultation numbers (ref. < 20)						
20–	–	–	1.41 (1.01–1.97)	–	–	–
≥ 40	1.81 (1.29–2.56)	–	1.99 (1.41–2.81)	2.52 (1.76–3.63)	–	2.46 (1.71–3.55)
Home visit (ref. Never)						
Occasion	0.46 (0.27–0.81)	0.42 (0.22–0.83)	0.45 (0.26–0.79)	–	–	–
Frequent	–	0.44 (0.23–0.83)	–	–	–	–
Occupational development opportunities (ref. Low)						
Middle	–	–	–	–	–	–
High	–	–	–	–	–	0.54 (0.32–0.91)
Work environment (ref. Bad)						
Fair	–	–	–	0.57 (0.40–0.79)	–	0.55 (0.40–0.77)
Good	0.58 (0.38–0.89)	–	0.57 (0.37–0.88)	0.65 (0.43–1.00)	–	0.65 (0.43–0.98)
Relationship between colleagues (ref. Bad)						
Fair	–	–	–	–	0.17 (0.03–0.81)	–
Good	–	0.23 (0.07–0.77)	–	–	0.09 (0.02–0.41)	–
Physician–patient relations (ref. Bad)						
Fair	0.52 (0.38–0.72)	0.52 (0.36–0.77)	0.51 (0.37–0.70)	0.43 (0.31–0.60)	0.38 (0.23–0.62)	0.39 (0.29–0.53)
Good	0.22 (0.14–0.35)	0.20 (0.12–0.36)	0.21 (0.13–0.34)	0.23 (0.15–0.36)	0.24 (0.13–0.45)	0.19 (0.13–0.28)
Practice environment (ref. Bad)						
Fair	0.62 (0.45–0.86)	–	0.61 (0.44–0.85)	0.68 (0.49–0.94)	–	–
Good	–	–	–	0.56 (0.33–0.95)	–	–

*WPV* workplace violence

^*^Includes those who experienced only physical, only non-physical, or both types of workplace violence

^a^Adjustment for practice setting (urban, rural), daily consultation numbers (< 20, 20–, ≥ 40), home visit (never, occasion, frequent), work environment (bad, fair, good), physician–patient relations (bad, fair, good), and practice environment (bad, fair, good), which were included in the final model during the stepwise process

^b^Adjustment for weekly working hours (≤ 40, 40–, > 50), home visit (never, occasion, frequent), relationship between colleagues (bad, fair, good), and physician–patient relations (bad, fair, good), which were included in the final model during the stepwise process

^c^Adjustment for practice setting (urban, rural), daily consultation numbers (< 20, 20–, ≥ 40), home visit (never, occasion, frequent), work environment (bad, fair, good), physician–patient relations (bad, fair, good), and practice environment (bad, fair good), which were included in the final model during the stepwise process

^d^Adjustment for practice setting (urban, rural), daily consultation numbers (< 20, 20–, ≥ 40), work environment (bad, fair, good), physician–patient relations (bad, fair, good), and practice environment (bad, fair, good), which were included in the final model during the stepwise process

^e^Adjustment for relationship between colleagues (bad, fair, good) and physician–patient relations (bad, fair, good), which were included in the final model during the stepwise process

^f^Adjustment for education level (associate’s degree or vocational diploma, bachelor degree, master degree or higher), practice setting (urban, rural), daily consultation numbers (< 20, 20–, ≥ 40), occupational development opportunities (fewer, general, more), work environment (bad, fair, good), and physician–patient relations (bad, fair, good), which were included in the final model during the stepwise process

## Discussion

This is the first national survey of the prevalence of WPV and its influencing factors among Chinese GPs. The survey showed that the prevalence of any type of WPV, non-physical violence, and physical violence was 14.26%, 14.03%, and 5.39%, respectively, and the prevalence varied between sexes.

Compared with the previous studies about WPV among GPs in China [6, 7, 21, 22], this study further expanded the findings of previous reviews in several important aspects. In the previous studies in China, the study population was selected by stratified random sampling, but was limited to a single province or township institutions. However, our study has investigated differences in WPV between rural and urban areas, which was a significant factor associated with the prevalence of any type of WPV and non-physical violence in the current study. Besides, we observed sex differences in the factors associated with the three types of WPV.

This study showed that 14.26% of Chinese GPs reported exposure to WPV during the previous 12 months, which was lower than the prevalence rates found in western countries (e.g., Australia, Ireland, Turkey, and the United Kingdom) ranging from 49.5 to 82.8% [11, 12, 14, 16]. Nevertheless, sample size, the measurement tools, national healthcare policies, the prevention and control of COVID-19, and socio-cultural diversities in different countries may contribute to the differences.

As we observed in Table 4, perpetrators were more likely to be middle-aged or older people, which may be related to the professional characteristics of GPs. In China, the gatekeeper’s policy and hierarchical medical system were not fully performed in the general population strictly [25, 26]. Many middle-aged or older residents were prone to seek medical care from GPs, but the young generation, was more likely to seek medical help from specialists in higher-level hospitals [27].

The results of multivariable stepwise logistic regression analysis revealed some interesting findings. Firstly, GPs who had home-visiting occasionally were less likely to expose to any type of WPV and non-physical violence. This was different from the previous studies [28–30], which found GPs who made home visits had an increased chance of an abusive encounter and perception of violence. This could be explained by the close connection built between GPs and patients during home visits. Secondly, GPs who practised in urban settings had more risk of any type of WPV and non-physical violence, which was similar to the previous studies [31, 32]. Some previous studies have shown patients who were hard to get medical service and got inadequate services are more likely to perpetrate violence [33, 34]. However, urban hospitals and community healthcare centres faced a high volume of patient consultations every day due to the high population density that not all the patients can be served properly. Besides, studies have shown that both the education level and GPs diagnosis or treatment competence are lower in townships than in urban medical institutions. Consistently, it was reported that this lack of competence was associated with anger among patients and family members. Thirdly, from the stratified analysis, female physical violence was only influenced by interpersonal relationships. We reviewed studies and found that female GPs were more likely to feel apprehensive about WPV [17, 32], and that might be one reason for the positive effect of good interpersonal relationships. Finally, this study showed that fewer patient consultation numbers per day, improving the work environment, and maintaining good physician–patient relations can reduce the incidence of all three types of WPV.

## Strengths and limitations

This is the first investigation of the prevalence of WPV and the relevant determinants among GPs in China at the national level. Secondly, the large sample size significantly increased the statistical power to identify determinants influencing WPV among GPs. Thirdly, data collection through universal social networks has greatly improved the response rate of the questionnaire, which makes the survey results and process have promotion significance. Finally, the results of this study will contribute to the improvement of existing strategies to reduce WPV in China and provide valuable evidence on the topic for the international general practice research field.

Some limitations should be acknowledged in our research. First of all, this was a cross-sectional survey, and the causal relationship between variables cannot be established; therefore, further longitudinal studies on such relationship are needed. Secondly, the data were obtained through self-reported, and the respondents inevitably had recall bias, which may overestimate the outcome. Thirdly, potential factors (such as personality traits, training programs, and the supervisory system) for WPV among GPs were more than listed in the questionnaire, and we cannot identify all of them. Fourthly, the generalization of the data to other populations in China, particularly other provincial administrative regions with least GDP out of sample may be limited. Finally, the COVID-19 control and prevention may influence the measurement of work-related factors. Thus, the impacts of COVID-19 from the relationship between work-related factors and WPV against GPs were unavoidable, because it has not been considered in the questionnaire.

The information on WPV was collected using a standardized scale by self-reporting. Although the scale of WPV was relatively objective and specific, the prevalence could have been underestimated. As we only investigated currently on-post GPs, turnover for violence and absence due to injury or even murder in WPV may be reasons for under-reporting WPV. More objective measurement tools such as the WPV surveillance system are needed in the future.

## Implications for research and practice

This research is the largest-scale cross-sectional study at the national level, revealing the prevalence and relevant determinants of WPV among Chinese GPs. However, the following aspects can be improved. First, the problems of horizontal violence among GPs, such as bullying and discrimination, were not given attention in this study. In addition, additional prospective cohort studies need to be conducted to clarify the causal relationship between these associated factors and WPV.

For policymakers, this study found that good physician–patient relations, good work environments, and occasional home visits were of great significance to reduce the incidence of WPV. Good incentive policies and stronger legal protections were related to a lower incidence of WPV in China. Thus, it is necessary to consider these views in the health policy-making process.

## Conclusion

The prevalence of WPV among GPs in China is comparatively low compared with other countries. The most common type of violence is verbal abuse, followed by threats, verbal sexual harassment, physical assault, and sexual assault. The perpetrators are mainly patients and males, and the main reasons for WPV among GPs are unmet patient needs, long waiting times, and being dissatisfied with GPs’ service. GPs who are male, practising in urban areas, with more patient consultation numbers, and working overtime frequently are more likely to be exposed to WPV; however, GPs who are working in a good work environment, in a good practice environment, with good physician–patient relations, and have home-visiting occasionally can reduce the risk of experiencing WPV.

## Data Availability

Data may be made available by contacting the corresponding author.

## Abbreviations

CHCs: Community health centres
CIs: Confidence intervals
GPs: General practitioners
ORs: Odds ratios
SD: Standard error
SPSS: Statistical Package for Social Sciences
WHO: World Health Organization
WPV: Workplace violence
